# MR imaging findings of a patient with isolated intrahepatic splenosis mistaken for hepatocellular carcinoma

**DOI:** 10.1259/bjrcr.20150242

**Published:** 2016-09-29

**Authors:** Kulyada Somsap, Nittaya Chamadol, Attapol Titapun, Chawalit Pairojkul, Sakkarn Sangkhamanon

**Affiliations:** ^1^Department of Radiology, Faculty of Medicine, Khon Kaen University, Khon Kaen, Thailand; ^2^Department of Surgery, Faculty of Medicine, Khon Kaen University, Khon Kaen, Thailand; ^3^Department of Pathology, Faculty of Medicine, Khon Kaen University, Khon Kaen, Thailand

## Abstract

Splenosis refers to the autotransplantation of splenic tissue throughout different anatomic compartments secondary to trauma or splenic surgery. The liver is an uncommon location for splenic implants and imaging findings are often described as non-specific. We report MRI findings of a patient with a large liver mass that was first diagnosed as a malignant tumour but histopathology revealed that it was actually intrahepatic splenosis. The signal characteristics of this mass were low intensity on *T*_1_ and high intensity on *T*_2_ weighted images; arterial enhancement, which became more homogeneous in the later phases; and a relative hypointensity on the delayed phase images. Because a high level of awareness is necessary for making a correct diagnosis of this condition, one should consider the possibility of intrahepatic splenosis in a patient with a history of abdominal trauma or splenic surgery.

## Background

Ectopic splenic tissue can be divided into two subgroups; one is an accessory spleen, which is congenital, and the other is splenosis, which is an acquired condition. Splenosis refers to the autotransplantation of splenic tissue throughout different anatomic compartments. It is thought to result from a capsular tear, either traumatic or iatrogenic, occurring during splenic surgery, with subsequent spillage and seeding of splenic pulp that later grows into a mass.^1,2^

Regarding the mode of splenic tissue autotransplantation, there are two possible routes: direct implantation and haematogenous spreading. The first accounts for the majority of cases, while the latter is believed to be responsible for the implantation of splenic tissue in distant compartments.^1,3,4^ The incidence of splenosis may be underreported, as most patients are asymptomatic. The common locations for splenosis include the mesentery, omentum, peritoneum and peritoneal cavity.^1,5,6^ However, uncommon sites such as the thoracic cavity, gallbladder, skin and brain have also been reported.

Intrahepatic splenosis is referred to as the autotransplantation of splenic tissue in the liver. It is an uncommon condition and, when present, is usually mistaken for a neoplasm.^7–12^ The reported imaging findings, especially MRI, are scarce and often non-specific.^5,8–12^ Here, we report the MRI findings of a patient with an isolated intrahepatic splenosis that was mistaken for hepatocellular carcinoma and discuss the possible helpful features for making the correct diagnosis.

## Clinical presentation

A 51-year-old male who had undergone a splenectomy 20 years earlier, supposedly owing to thalassaemia, presented to another hospital with intermittent abdominal pain at the left upper quadrant. There was no history of weight loss or any other abnormal symptoms. A liver mass was found during the initial work-up and he was then referred to our hospital for further investigation and management.

## Investigations

The liver function test revealed slightly elevated aspartate transaminase, alanine transaminase and total bilirubin levels. The details were as follows: alanine transaminase 64; aspartate transaminase 75; alkaline phosphatase 64; total bilirubin 1.7 and direct bilirubin 0.3; albumin 3.8; globulin 2.3. The hepatitis B and C profiles were negative. Tumour markers, including cancer antigen 125, alpha-fetoprotein, cancer antigen 19-9 and carcinoembryonic antigen, were all negative.

Ultrasound images from other hospitals were not available for interpretation.

**Figure 1.**
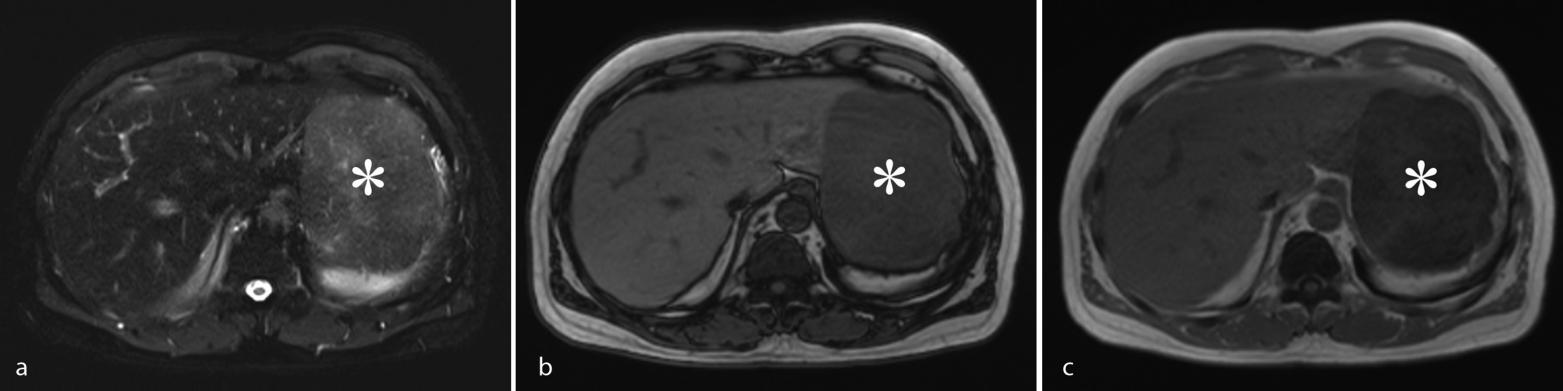
MRI of the liver shows a large mass (asterisk) at the left lobe with high signal intensity on *T*_2_ weighted image (a) and low signal intensity on opposed-phase *T*_1_ weighted image with significant signal dropout on in-phase *T*_1_ weighted image, which is representative of haemosiderin deposition (b, c).

**Figure 2.**
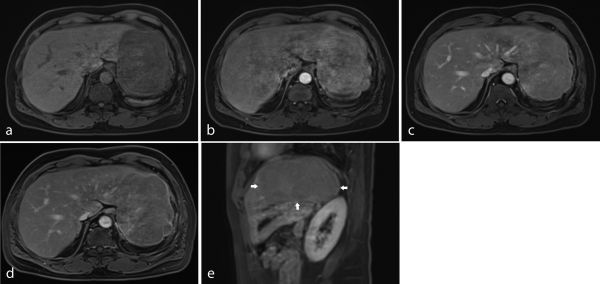
Pre-contrast *T*_1_ weighted fat-suppression image shows a hypointense mass at the left hepatic lobe (a). Dynamic contrast-enhanced MRI shows heterogeneous enhancement of the mass at the left hepatic lobe in the arterial phase (b). The mass becomes more homogeneous on portal venous and delayed images (c, d). Sagittal image acquired during the delayed phase shows a homogeneous mass (outlined by arrows), which appears relatively hypointense to the liver parenchyma (e).

**Figure 3.**
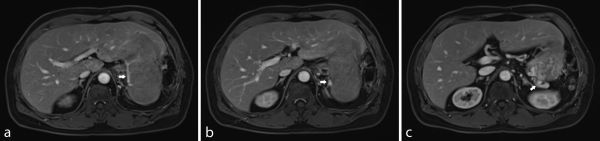
Images show extrahepatic venous drainage (arrows) of this mass into the gastric varices, which ultimately drains into the portal venous system *via* the left gastric vein. The presence of isolated gastric varices may be explained by the impaired venous drainage from previous ligation of the splenic vein (a–c).

MRI showed a large, slightly lobulated exophytic mass at the left lobe of the liver without a separable fat plane between the mass and the liver parenchyma. The signal intensity of this mass on *T*_1_ and *T*_2_ weighted images is shown in Figure 1, while the enhancement characteristics are shown in Figure 2. This mass appeared slightly hyperintense on diffusion-weighted images. Extrahepatic venous drainage was noted (Figure 3). The spleen was absent. In addition, there was no mass elsewhere in the abdomen.

## Diagnosis and treatment

**Figure 4.**
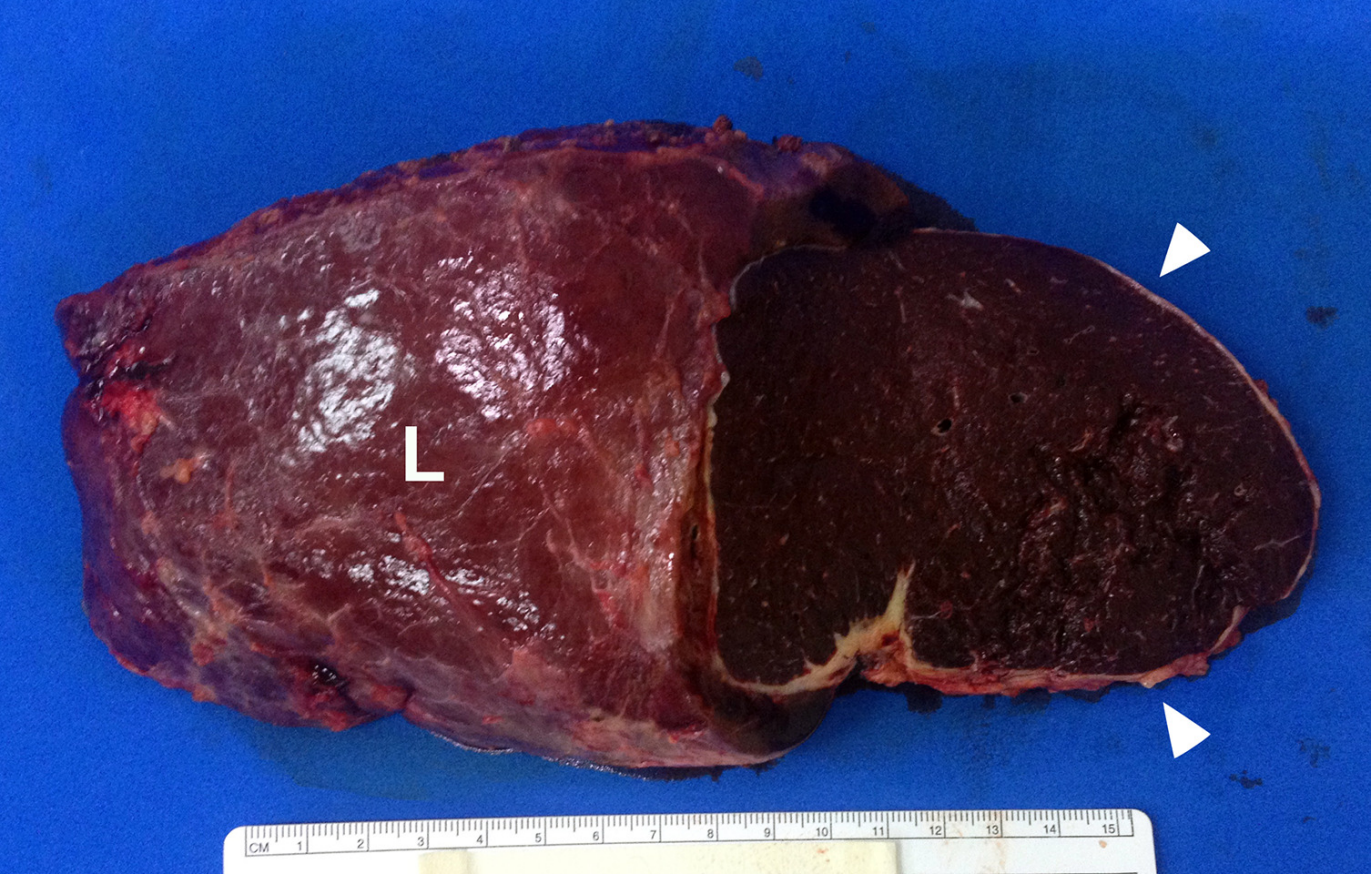
The gross specimen shows a large mass (arrowheads) in the lateral segment of the left hepatic lobe (L) with a cut surface similar to that of the spleen.

**Figure 5.**
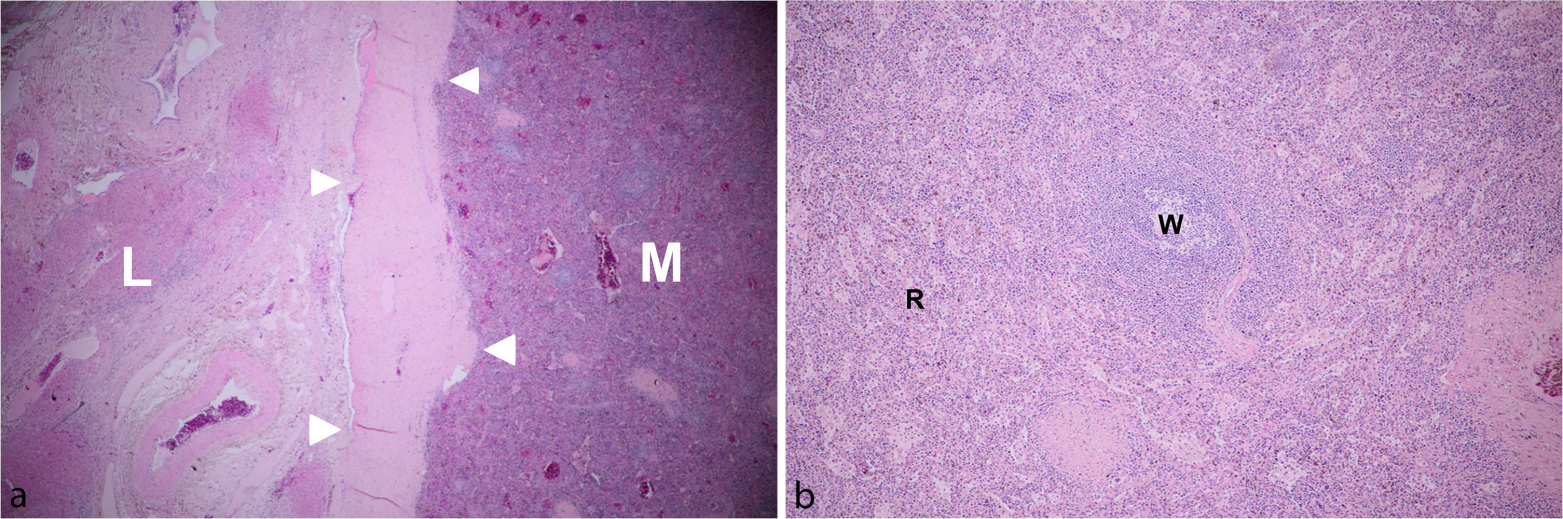
Microscopic examination (low power field, 4×) shows the interface between a well-encapsulated mass with thick fibrous capsule and adjacent liver parenchyma (a), containing red and white pulp (b). (L, liver; M, mass; R, red pulp; W, white pulp; arrowheads, fibrous capsule).

Owing to its large size, the mass was presumed to be malignant. The pre-operative diagnosis favoured hepatocellular carcinoma because of the arterial-phase enhancement and the delayed-phase hypointensity. The patient subsequently underwent a left lateral segmentectomy, from which the gross specimen revealed a mass within the left hepatic lobe. However, the cut surface of this mass looked similar to that of the spleen (Figure 4) and the histopathology turned out to be splenic tissue embedded in the liver parenchyma (Figure 5). Thus, the final diagnosis of intrahepatic splenosis was made.

## Discussion

Isolated intrahepatic splenosis is rare. Although it is a benign condition, diagnosing it can be difficult in clinical practice because it can mimic a liver tumour, which frequently leads to unnecessary surgery.

Currently, there are a number of hypotheses regarding the mechanism of hepatic implantation of splenic tissue, including invagination or exophytic growth of splenic tissue that is directly seeded into the liver capsule, and haematogenous spreading due to entry of an erythrocyte progenitor cell into the portal venous system.^3,13,14^

Imaging findings of splenosis have been often described as non-specific. Most authors described the MRI characteristics of splenosis as low signal intensity on *T*_1_ and iso- to high signal intensity on *T*_2_ weighted images.^5,8,10,11,15^ Because of the difference of blood flow in the red and white pulp, the splenic implants were shown to have heterogeneous enhancement on the arterial phase and became homogeneous in the later phases of dynamic MRI.^5,8,15^ On the delayed phase, the signal intensity of intrahepatic splenosis may be lower than that of the liver parenchyma.^9^

A previous study on the apparent diffusion coefficient value of intra-abdominal organs has shown that the value was lowest in the spleen.^16^ As in a normal spleen, restricted diffusion has also been demonstrated in the case of splenosis.^17^

The signal characteristics and enhancement pattern of intrahepatic splenosis in our case are similar to those previously reported—that is, being hyperintense on *T*_2_ and hypointense on *T*_1_ weighted images, with heterogeneous enhancement on the arterial phase and becoming more homogeneous in the later phases. Diminished enhancement was also observed on delayed images. Diffusion-weighted images showed a slightly high signal intensity of the lesion.

Inchingolo et al^8^ reported a signal dropout on in-phase *T*_1_ weighted image when compared with the signal on the opposed-phase images, which was indicative of haemosiderin deposition in splenic tissue. Likewise, this finding was also observed in our study. Thus, we believe the presence of a signal change in chemical-shift imaging to be a helpful feature for the diagnosis of splenosis.

Moreover, the demonstration of extrahepatic venous drainage is unusual for a primary liver tumour. The presence of such vessels should thus raise the suspicion of another possible diagnosis.

As described in a previous report, a thin rim of fat at the border of the lesion was not visualized in our study.^10^

Above all, a high level of awareness remains the most crucial factor in the diagnosis of splenosis. If recognized preoperatively, one can opt for non-invasive imaging confirmation such as ^99m^Tc denatured red blood cell scintigraphy or liver MRI using superparamagnetic iron oxide-based contrast agents, and avoid unnecessary surgery.^10,14,15^ Therefore, in the case of patients with a history of splenic surgery or abdominal trauma, one should consider splenosis in the differential diagnosis.

## Learning points

1. In the case of patients with a history of splenectomy or splenic trauma, the presence of an arterially enhancing liver mass that becomes more homogeneous in the later phases should raise the clinical suspicion of splenosis.
2. The presence of a signal dropout on in-phase images and extrahepatic venous drainage are helpful features for the diagnosis of this pathology.
3. If splenosis is suspected, there are non-invasive imaging modalities available for the confirmation of this pathology.

## Ethical considerations

This retrospective case report was approved by the Ethic Committee for Human Research based on the declaration of Helsinki and the International Conference on Harmonization good clinical practice guidelines. Clinical data were obtained by reviewing medical records. MRI findings were reviewed and consented to by two radiologists specializing in gastrointestinal and hepatobiliary imaging.

## Consent

Informed consent for the case to be published was obtained for publication of this case report, including accompanying images.
